# Innovations in Women’s Bone Health—Appreciating Important “Bone Variables” Besides Estrogen

**DOI:** 10.3390/ijerph15091929

**Published:** 2018-09-05

**Authors:** Jerilynn C. Prior

**Affiliations:** 1 Division of Endocrinology and Metabolism, Department of Medicine, University of British Columbia, Vancouver, BC V5Z 1M9, Canada; jerilynn.prior@ubc.ca; Tel.: +1-604-875-5927; 2 Centre for Menstrual Cycle and Ovulation Research, University of British Columbia, Vancouver, BC V5Z 1M9, Canada; 3 School of Population and Public Health, University of British Columbia, Vancouver, BC V6T 1Z3, Canada; 4 BC Women’s Health Research Institute, Vancouver, BC V6H 2N9, Canada

We learned in 1969, the year I graduated from medical school, that—based on the new technique of quantitative microradiography of an iliac crest bone biopsy—increased bone resorption characterized osteoporosis in both women and men as diagnosed by fragility vertebral fracture [1]. Conjugated equine estrogen treatment in the 11 women and testosterone treatment in the single man studied led to significantly decreased bone resorption documented by decreased percentage of crenelated bone surfaces [1]. As would be expected based on coupling of resorption and formation, both estrogen and testosterone also decreased bone formation in 10 of 12 participants as assessed by the extent of tetracycline labelling [1]. Almost 50 years later, we can now reasonably accurately predict 10-year risk for fragility fracture based on clinical risk factors with or without measuring areal bone mineral density (BMD) [2]. We have also learned that hip fractures are not just an “old woman’s disease” since population-based older men surviving in the community to age ≥ 75 years have similar 10-year hip fracture risks (~7%) as women aged 75 or older in the same population [3]. However, we have not yet accomplished effective prevention of fragility fracture nor perfected osteoporosis therapy that both *decreases* bone resorption AND *increases* bone formation. 

As in the above study from almost five decades ago, women’s bone health still tends to focus on estrogen, to designate women as osteoporosis victims and to assign the reason for this vulnerability to the “estrogen deficiency” of menopause. Logic says that concept should be well refuted—all women become menopausal, menopause always is characterized by low estradiol and progesterone levels yet not all women become osteoporotic. However, estradiol that indirectly suppresses bone resorption and progesterone that stimulates osteoblastic bone formation [4] are both important components of the menstrual cycle; further, a fertile cycle requires both normal estradiol and normal ovulation with sufficient progesterone. Furthermore, osteoporosis treatments continue to primarily act through antiresorptive mechanisms (such as their archetype, estrogen) while ignoring the fact that to suppress bone resorption is also to suppress bone formation through the coupling of these processes [5].

These narrow concepts and limited approaches ignore the fact that bone remodeling is a complexly *integrated system* with two major strongly-linked processes at its core, bone resorption and bone formation. The important, but often ignored, differentials in timing of completed resorption and completed formation within each bone mineralizing unit—three weeks for resorption and three months for formation—also encourage the focus on antiresorptive therapies that therefore take a dominant role. Thus bone resorption is fast and bone formation is slow; that fact does not imply, however, that **both** processes are not essential. 

This Special Issue on “New Concepts in Women’s Bone Health” illustrates some of the breadth of biochemical, clinical, epidemiological and sociocultural variables that are relevant to women’s osteoporosis prevention. This Issue included work from scientists in five countries and presented an incredible array of perspectives/topics. These bone-relevant variables included shorter leukocyte telomere lengths related to lower BMD in women living with HIV [6], insulin resistance related to reduced bone strength in menopausal Chinese women without diabetes [7] and altered polar lipid metabolomics associated with lower femoral neck BMD [8]. 

Other bone-relevant topics included population-based data on BMD and prevalent fractures in premenopausal women [9]. These data showed that lower lumbar spine BMD related to amenorrhea, as expected, but also surprisingly to medically important androgen excess for which participants sought physician treatment [9]. This is the first time that epidemiological data have clearly linked potential bone health risk to women with Polycystic Ovary Syndrome (PCOS) who tend to weigh more, who have higher androgen, definitely normal or high estrogen levels despite oligomenorrhea, and often higher insulin levels and have thus been thought to be protected from osteoporosis [9]. In addition those same data in the Canadian Multicentre Osteoporosis Study (CaMos) showed that a later age at menarche was associated with lower femoral neck BMD while both body mass index and height were positively related to BMD at the lumbar, femoral neck and total hip sites [9]. Unfortunately the presence of molimina (normal premenstrual symptoms) is not diagnostic of a hormonally documented ovulatory cycle [10]. The problem is that anovulation is silent within regular menstrual cycles [11] and, in meta-analysis, those women with more versus less prevalent silent ovulation within regular menstrual cycles were losing −0.86% more spinal BMD/year [12]. In addition, the population-based CaMos investigation, midlife women showed that those with more intense/frequent night sweats tended toward increased two-year BMD loss [13]. 

Medication use may both have different effects in the two sexes and also be differently utilized based on gender. Another article in this Special Issue asked whether BMD was related to a history of anti-depressant or anti-anxiety therapy [14] given that use of serotonin reuptake inhibitors has been repeatedly related to increased fracture risk in population data [15,16]. On the theme of prevention of osteoporosis, Troy et al. reviewed how and why physical activity variables are highly relevant to areal BMD, volumetric BMD and strength across women’s life cycles [17]. Finally, in women who had experienced a fragility fracture and been referred to a fracture liaison programme, whether or not women believed their fracture was related to osteoporosis made a difference in whether they adhered to the programme’s recommendations for exercise, vitamin D and calcium intakes [18]. 

Thus the horizons of women’s bone health have expanded beyond fragility fracture—this elderly women’s pre-death catastrophe—into opportunities for fracture prevention. This is based on a newly emerging concept that it may be wise to avoid use of combined hormonal contraceptives in adolescent women [19] whose bones need freedom for bone modeling as they gain toward their peak BMD [20]. It also relies on simple lifestyle choices such as a commitment to life-long healthy eating [21], habitual, everyday physical activity [22], avoiding obesity and insulin resistance [23] and skills and support to deal with the subtle life stresses that lead to chronically silent ovulatory disturbances. It still remains to be shown, however, that chronic ovulatory disturbances within regular cycles are related to increased fracture risk as well as to documented loss of spinal BMD [12]. Together there are various, simple ways to achieve effective osteoporosis prevention as summarized for women of different life phases (www.cemcor.ca/resources/abcs-osteoporosis-prevention-premenopausal-women). 

There is a strong need now to also think about osteoporosis therapy as interacting with imbalances in the bone remodeling system to both prevent rapid bone loss and to promote increased bone formation. Such synergism of therapy with estradiol/estrogen plus progesterone/progestin has been shown to exceed the BMD benefit of estrogen alone in a meta-analysis of randomized controlled trials [24] and in a RCT of parathyroid hormone based therapy followed by an antiresorptive versus each individual therapy alone [25]. Finally it remains to be documented that such combined, synergistic therapy will improve fragility fracture prevention more effectively than antiresorptive therapy or bone formation-stimulating therapy alone. However, this will likely soon be documented, as further new concepts arise in women’s bone health.
